# How to Approach Left Ventricular Hypertrabeculation: A Practical Guide and Literature Review

**DOI:** 10.3390/jcm14030695

**Published:** 2025-01-22

**Authors:** Michele Alfieri, Samuele Principi, Alessandro Barbarossa, Giulia Stronati, Roberto Antonicelli, Michela Casella, Antonio Dello Russo, Federico Guerra

**Affiliations:** 1 Department of Biomedical Sciences and Public Health, Marche Polytechnic University, 60131 Ancona, Italy; m.alfieri@inrca.it (M.A.); giulia.emily.stronati@gmail.com (G.S.); michela.casella@ospedaliriuniti.marche.it (M.C.); antonio.dellorusso@ospedaliriuniti.marche.it (A.D.R.); 2 Cardiology Unit IRCCS INRCA, Via della Montagnola 81, 60127 Ancona, Italy; r.antonicelli@inrca.it; 3 Cardiology and Arrhythmology Clinic, Department of Cardiovascular Sciences, Marche University Hospital, 60126 Ancona, Italyalessandro.barbarossa@ospedaliriuniti.marche.it (A.B.); 4 Department of Medical and Surgical Sciences, Marche Polytechnic University, 60131 Ancona, Italy

**Keywords:** cardiomyopathy, left ventricular hypertrabeculation, excessive trabeculation, left ventricular non-compaction, heart failure, sudden cardiac death, ventricular fibrillation, ventricular tachycardia

## Abstract

Left ventricular hypertrabeculation is one of the most debated conditions in modern cardiology. Many studies have tried to characterise this disease by addressing the various clinical risks and diagnostic tools, but its very nosological existence is currently being challenged. The latest ESC guidelines on cardiomyopathies state that it should be addressed as a morphologic trait rather than an intrinsic disease of the cardiac muscle. Despite the huge number of diagnostic criteria and possible phenocopies, no specific consensus identifies a specific flowchart regarding the management of patients with suspected hypertrabeculation. This review aims to provide a clinical approach for patients with a phenotypical appearance of excessive trabeculation.

## 1. Introduction

Left ventricular hypertrabeculation (HT), previously named “non-compaction”, is a relatively misunderstood and neglected condition lying on the edge between a full-fledged cardiomyopathy and a benign variant of the cardiac anatomy. It is characterised by the presence of prominent trabeculae and deep intertrabecular recesses communicating with the ventricular cavity. For years, it has been considered a separate disease (for some, the third most common cardiomyopathy) carrying serious clinical risks, namely, cardioembolism, heart failure (HF), and arrhythmias. It must be differentiated from other excessive trabeculation patterns, where some diseases or even physiological conditions may induce this morphological variant without the typical clinical implications of HT cardiomyopathy. This overlap with “benign” excessive trabeculation has led the latest ESC guidelines to challenge its classification as a full-fledged cardiomyopathy by opening a debate on whether it should only be labelled as a simple phenotype [1]. This review aims to clarify what has been achieved in the understanding of this condition while providing a practical clinical approach to patients with excessive trabeculation.

## 2. Organogenesis

During embryogenesis, endocardial cells give rise to the trabecular myocardium in both the atria and ventricles. The distribution and prominence of trabeculae in the ventricular chambers are only partially mediated by a genetic phenomenon since a major role seems to be dictated by differential blood flow in every single segment [2]. Lower shear stress on the apex might then be the key to understanding why this region frequently hosts the most trabeculae. Several studies have shown how trabecular myocardium carries different molecular signalling pathways than compact myocardium, thus providing different functions. First of all, trabeculae, through a wider surface, can grant oxygen and nutrient exchange before coronary development; secondly, the differential connexin expression in this region allows a more efficient transmission of electrical impulse and marks the future formation of the Purkinje fibres [3,4]. In some species, this internal layer undergoes a process of compaction, leading to the development of the normal muscular walls; in humans, this process has never been demonstrated, and from recent studies, it seems that trabeculae do not turn into a compact ventricular wall [4]. On the contrary, they evolve into different structures instead: in the atria, they give rise to pectinate muscles, while in the ventricles, they participate in the formation of papillary muscles, atrioventricular valves, and, as mentioned before, Purkinje fibres [4,5]. Thus, the term “non-compaction”, deriving from the old belief that non-compacted myocardial structures were transient and progressively folding into the compact ventricular layers, has been abandoned [5]. In most adults, the predominance of the compact ventricular walls can be explained by the concept of “allometric growth”. This model states that different structures can grow at a different pace depending on the very specific stage of development, exactly like trabecular and compact myocardium, which form during different phases and undergo separate growth curves [5,6,7]. In fact, during embryogenesis, trabeculae have an initial stage of rapid maturation that soon slows down around the fifth week of gestation, letting the compact walls take over the majority of myocardial structure; in some instances, this process can be disrupted, and a trabeculated phenotype may persist after development.

## 3. Genetic Considerations

There is no univocal genetic pattern justifying the origin of HT cardiomyopathy. Previous studies have found several mutations in different genes responsible for a plethora of intracellular pathways. Among these, Notch1 is one of the most frequently involved, and via interactions with other proteins such as BMP10 and FKBP-12, it regulates trabeculae formation during the early stages of development. Notably, knockout mice for some of these genes die prematurely in utero [3,8]. SCN5A defects have also been described; this gene codifies for a sodium channel whose mutations are responsible for several diseases; in HT cardiomyopathy, it has been described to develop a phenotype prone to arrhythmic manifestations and HF [9]. A particular mutation in the G4.5 (previously known as TAZ) gene, encoding for a protein known as tafazzin, is responsible for a rare X-linked disease known as Barth syndrome, characterised by growth retardation, neutropenia, and, most importantly, skeletal myopathy and cardiomyopathy [10]. The cardiac phenotype is present in 70% of the affected patients [11], most frequently involving the left ventricle with a dilatative and HT pattern. The most important clinical features are HF and arrhythmias with the need for heart transplantation in up to 12% of the patients affected [10,11]. From our perspective, the various genotypes related to this condition might represent a red flag but, at present, should not be used as a diagnostic tool.

## 4. Clinical Manifestations

**Figure 1:**
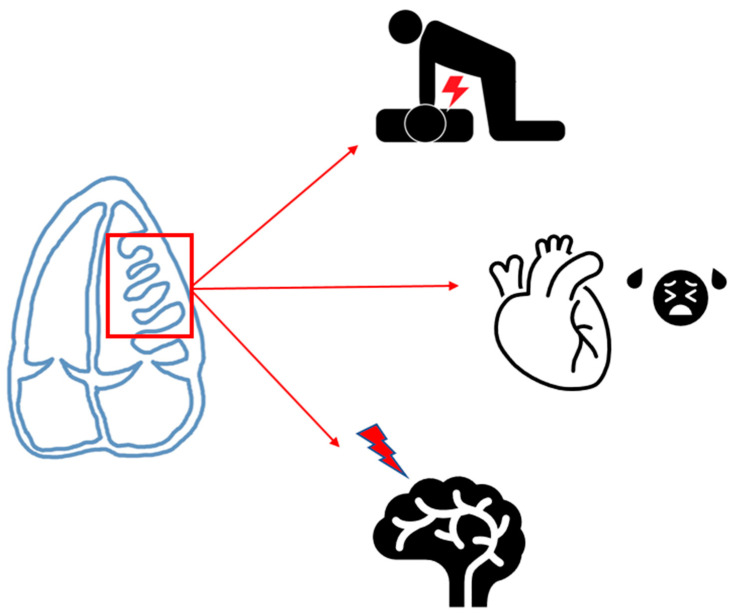
A schematic representation of the three major clinical complications of HT; from top to bottom: ventricular arrhythmias, heart failure, and stroke.

**Figure 2:**
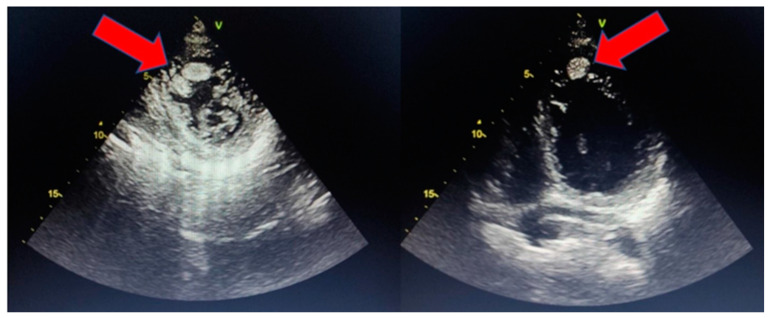
Endoventricular thrombosis in a 48-year-old male patient with HT cardiomyopathy admitted to our cardiology department due to new-onset heart failure; the thrombus was successfully treated with intravenous heparin. The arrows indicate the thrombus inside the ventricular cavity.

The three major risks related to HT are ventricular arrhythmias, ventricular thrombosis, and HF (Figure 1). Several studies have found a higher incidence of ventricular arrhythmias in patients with HT; some registries report incidences of ventricular arrhythmias of 18–47%, with sudden cardiac death (SCD) ranging up to 18% [12]. Three proposed mechanisms for such events are interstitial fibrosis, microvascular dysfunction, and abnormal intercellular coupling. Interstitial fibrosis is the trigger for re-entry tachycardias, in which the presence of circuits, “protected zones”, and slow-conducting channels create an encircling mechanism where the electrical impulse can travel in a loop and perpetuate. In addition, microvascular dysfunction may induce ischaemia in some areas, thereby triggering post-potential activities. On the other hand, no clear evidence exists regarding the role of altered Purkinje fibres in this setting [13,14,15]. There is no clear evidence regarding the prevalent physiopathological explanation for ventricular arrhythmias, but a probable rationale could be an overlap between these mechanisms starting from a microvascular dysfunction directly leading to an increase in the rest of the membrane potential of an already-damaged Purkinje system and to focal fibrosis predisposing to re-entry phenomena. Endocavitary thrombosis is another threatening complication leading to embolic events like stroke (Figure 2); its prevalence ranges up to 24% in adults and 38% in children, although with significant variability in different studies [12].

Unlike thrombosis occurring in ischaemic patients, it is not related to a segmental contraction deficit of the ventricular wall but more likely to the relative blood stasis between trabeculae, which do not have contractile properties. Some evidence suggests that this predisposition could be also enhanced by microvascular dysfunction and the overexpression of pro-coagulant factors, thus satisfying the criteria of the Virchow triad [16,17]. Further evidence confirms such a hypothesis, suggesting that a higher non-compacted (NC) volume might be directly related to the risk of stroke [18]. Lastly, HF is the third clinical pillar in patients with HT with an incidence of up to 76% in adult patients. The main aetiological theory lies in the presence of reduced perfusion due to the underlying microvascular dysfunction on top of genetic and structural abnormalities [12,15]. A reduction in myocardial perfusion has been demonstrated by PET studies [19], which have shown, even in children, a reduced coronary blood flow and subendocardial fibrosis despite normal epicardial coronary arteries; this could probably be the reflection of an underlying microvascular dysfunction, although no certain conclusions can be drawn on the matter. It is important to point out that the aforementioned clinical presentations do not occur in a considerable number of patients, and valid predictors for identifying individuals at risk of adverse events are still lacking.

## 5. Imaging

A detailed dissertation of the different diagnostic criteria is beyond the scope of this review. Several methods have been used to identify the disease, and every one of them focusses on trabecular prominence, and echocardiographic criteria are slightly different from one another. The original one proposed by Jenni takes into account the ratio between NC and compacted (C) thickness in end-systole (ratio > 2) with blood flow communicating with trabecular recesses. Stöllberger refers to the presence of at least three trabeculations with intertrabecular blood flow in the area between papillary muscles and the cardiac apex. A further condition is an end-diastolic NC/C ratio ≥ 2. Lastly, Chin parameters consider the end-diastolic C/(NC + C) ratio in the parasternal long-axis or the apical four-chamber view with a diagnostic value ≤ 0.5 [20,21,22]. Cardiac magnetic resonance (CMR) is the other pivotal diagnostic and prognostic tool for HT patients, and even in this case, the most widely used criteria carry the name of the proposing authors. Petersen’s criteria are quite simple and consider an NC/C ratio >2.3 in diastole as diagnostic. Later, Stacey proposed an end-systolic NC/C ratio >2. In contrast with these methods, some authors have focussed their attention not only on the mere thickness of both layers but rather on the concept of “trabecular mass”. According to Jacquier, the diagnosis of HT can be confirmed if a trabecular mass exceeds 20% of the total ventricle, while Grotohoff adopts a more stringent cutoff with an NC mass >15 g/m^2^ and >25% [23,24,25]. To date, despite the anecdotal evidence showing the higher efficiency of some criteria among the others, there is no clear evidence regarding which one should be chosen and, most importantly, if any of these methods are able to identify a clear pathological condition. In clinical practice, an echocardiogram is much faster and more feasible than a CMR and represents the exam of choice in carriers of an implantable device due to the frequent artefacts occurring in these cases. On the other hand, CMR is the gold standard for patients with a poor echographic window and, most importantly, provides further insights regarding myocardial structure and composition and, consequently, prognosis; the presence of late gadolinium enhancement is a strong predictor of adverse outcomes [26]. According to Towbin [27], the presentation of HT can be multi-phenotypic with some pivotal patterns: arrhythmic, hypertrophic, mixed (hypertrophic-dilated), restrictive, right- or biventricular, with congenital anomalies, and benign. This wide expression puts even more variability into both ecographic and CMR criteria, thus implying the need for an open application of both techniques. Not by chance, the benign variant seems to be the most common, accounting for 37% of all cases; the real problem with this variant is to then identify the effective presence of a primary myocardial disease or, conversely, a secondary condition able to induce a form of increased “bystander” trabeculation.

## 6. Phenocopies

Echocardiograms and CMR are the most solid ground on which diagnosis is built, but unfortunately, imaging criteria can be inaccurate. Studies have shown that meeting one or more criteria does not necessarily mean that a clear HT diagnosis is present. There are benign conditions (“phenocopies”) resembling an HT pattern without any underlying pathological condition. The first example is the athlete’s heart. In athletes, an increase in cardiac preload due to high exercise volume can induce a progressive harmonic ventricular dilatation, and in up to 8% of cases, it can be accompanied by a more pronounced trabeculation that can reach the diagnostic criteria. Unlike patients with suspected cardiomyopathy, in these cases, the phenotype tends to recede after a de-training period [28,29,30]. Another example is pregnancy. In this condition, blood volume can double itself, thereby inducing a rapid increase in cardiac output, preload, and left ventricular volume, leading to the formation of a trabecular pattern [30]. A study conducted by Gati et al. showed that, by the third trimester, 8% of women developed a significant ventricular trabeculation meeting both the Jenni and Chin criteria, although CMR imaging was not applied [31]. Even some pathological conditions can be responsible for a non-compaction-like appearance: the most immediate example is aortic regurgitation, where a preload increase is the pathological mainstay of the disease [7]. Furthermore, even some extracardiac diseases may induce the formation of trabeculae, such as haematological (e.g., sickle cell anaemia) and renal disorders [7,32,33]. Starting from here, every cardiologist must bear in mind that not every trabecular pattern is necessarily the reflection of an intrinsic myocardial disease, but a hint needs to be taken into account before jumping to conclusions [32,34].

## 7. Diagnosis

**Figure 3:**
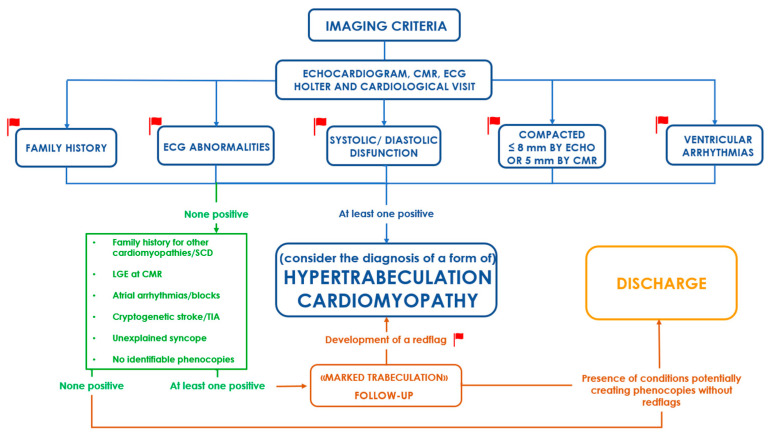
A practical approach to patients with excessive trabeculation. Red flags represent the features leading to the final diagnosis, while additional clues (in green) may be used to keep the patient under follow-up in the absence of a clear red flag.

According to what was discussed so far, it is now clear why the presence and recognition of HT as a pathological condition are still controversial. In Figure 3, we propose a diagnostic algorithm currently used in our centre to identify patients at higher risk of events.

**Figure 4:**
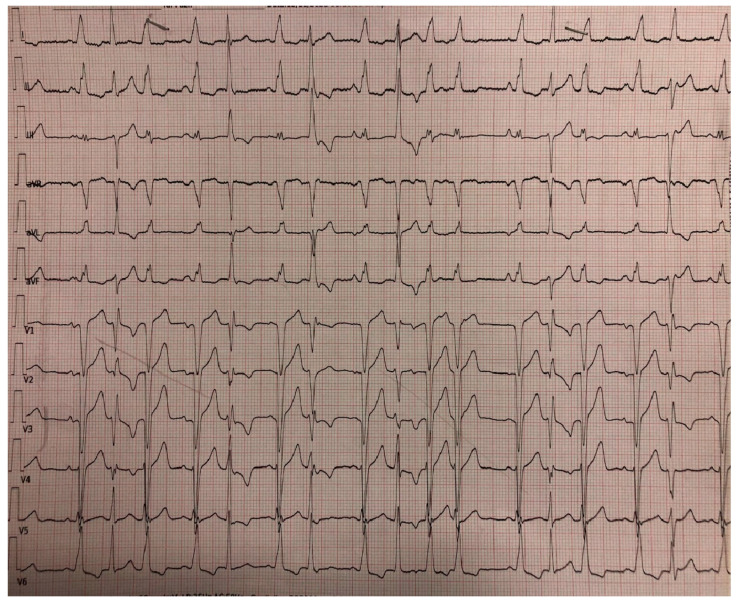
An ECG of a 62-year-old woman with heart failure and an HT phenotype. Note the frequent ectopies and the pre-excitation-like appearance.

Not every item carries the same weight, and real diagnostic power is often difficult to establish. First of all, the presence of symptoms (e.g., syncope, dyspnoea, and palpitations) or a family history of HT may be the first hint that could prompt a deeper investigation. Secondly, ECG abnormalities (fragmented QRS, extrasystoles, long QTc, inverted T waves, and complete bundle branch block) might be an important clue reflecting an electrical instability or even a structural underlying condition (Figure 4); anomalies in the ECG occur in up to 94% of patients affected by HT [12], and more generally speaking, a normal ECG is a rare occurrence in patients affected by any kind of cardiomyopathy [1].

Additionally, ventricular arrhythmias should always prompt further investigations considering their high rate in patients with HT cardiomyopathy and, most importantly, their heavy prognostic impact [13]. For the same reason, every unexplained syncope in such patients should always be investigated, even with the help of an implantable loop recorder if necessary. Echocardiographic alterations (systolic or diastolic dysfunction, a reduced thickness of the compacted layer) are another red flag. A study from Gebhard et al. [35] found that a reduced thickness (namely, <8 mm) of the compact layer is specific for the diagnosis of HT; such data have also been confirmed by more recent CMR studies, which found how a thin compact layer thickness is associated with a reduction in left ventricular systolic function, probably due to the relatively lower contractile capabilities of these elements [36]. This finding might be an indicator of the fact that, even if the growth rate and patterns of NC and C layers are different, they are strictly correlated.

Further relevant information could be the occurrence of a stroke of unknown origin (ESUS) in the absence of atrial fibrillation or carotid stenosis (especially in patients where a ventricular thrombus can be detected). To check for all these elements, a fundamental role is covered by the CMR. Apart from its diagnostic power held by the applicability of the aforementioned criteria, this tool can reveal other findings useful for identifying a complication that may aid a proper characterisation. For example, in cases where echocardiography is limited by the acoustic window or by the excess of trabeculae, CMR might be able to reveal the presence of a ventricular thrombus [37]. Furthermore, it can precisely measure the thickness and composition of each layer, and through the presence of late gadolinium enhancement (LGE), it can detect an underlying myocardial disease that would otherwise go undiagnosed. The sole presence of LGE is not a diagnostic parameter of HT, but it reflects the presence of a pathological substrate, thereby providing additional prognostic information [26,38]. Of course, not every red flag can be easily detected; for this reason, in patients with a clinical suspicion or a typical echographic pattern, further diagnostic exams should be always performed. In our centre, in addition to a cardiological visit with ECG and an echocardiogram, we usually perform a CMR and an ECG Holter. Any of the above pathological findings is then the starting point for a structured follow-up.

## 8. Therapy

The 2022 ESC guidelines on ventricular arrhythmias [39] and 2021 ESC guidelines on heart failure [40] do not provide specific indications for HT, comparing it to other conditions like dilated, hypokinetic non-dilated, or even hypertrophic cardiomyopathy. To date, the only tailored recommendations come from a 2019 HRS consensus statement [41], indicating the need for ICD implantation in patients with previous ventricular arrhythmias with syncope or resuscitated SCD; in addition, an ICD is suggested in patients with a reduced ejection fraction (EF) and non-sustained ventricular tachycardias. Other studies have also assessed the efficacy and safety of trans-catheter ablation in HT patients [14,42], and interestingly, a high percentage of ventricular arrhythmias tend to originate from heart segments without trabeculae. Furthermore, according to the same document, anticoagulation should be initiated without delay in patients with atrial fibrillation (AF) or known embolic events, regardless of CHADS-VA score, and might be considered in patients with a reduced EF without AF. A similar opinion is expressed by an American Heart Association scientific statement [43], which does not support the routine use of anticoagulation therapy, but it may be considered in patients with a history of stroke/TIA or with systolic dysfunction. Embolic risk has also been addressed by another paper from Chimenti et al. [44] suggesting the possibility of beginning anticoagulation for patients with a CHADS-VA score ≥ 2 without AF. There is no specific recommendation regarding pharmacotherapy for HF, and in our opinion, the four pillars (namely, beta-blockers, SGLT2 inhibitors, angiotensin receptor blockers/angiotensin receptor neprilysin inhibitors, and mineral corticoid receptor antagonists) should be adopted in the absence of contraindications. A promising tool is represented by device therapies; a study from Bertini et al. [45] analysing a group of patients with dilated cardiomyopathy, QRS ≥ 120 ms, and an HT phenotype showed the beneficial effects of cardiac resynchronisation therapy (CRT), and such benefit was more evident in patients with a higher representation of NC segments at baseline. In addition, the group of patients with HT had a higher incidence of responders and super-responders; this is in line with previous studies suggesting a more efficient contractile response in segments affected by HT [46], although the underlying pathophysiological mechanism is still obscure.

## 9. Sports Eligibility

The presence of prominent trabeculations is often attributable to a physiological adaptation to physical exertion, and thus, the sole presence of imaging criteria should not be used to diagnose HT cardiomyopathy [47]. On the other hand, studies have found that athletes with a real HT cardiomyopathy often present with symptoms, mainly syncope (60%), but also embolic events and, in some cases, even SCD [48]. The same studies also demonstrated that the main ECG abnormality in such patients is the presence of criteria for left ventricular hypertrophy, but some individuals may present with ventricular arrhythmias (from extrasystole to sustained ventricular tachycardias) or even atrial fibrillation [48,49]. The 2023 Italian guidelines for sports participation (COCIS) [46] identify some high-risk criteria for patients with HT, namely, symptoms (mainly syncope), a family history of ventricular arrhythmias, documented arrhythmias (atrial tachyarrhythmias or complex ventricular ectopy), LGE detection at CMR, and left ventricular dysfunction. In the presence of at least one of these criteria, competitive sports eligibility should be denied, while it might be considered for patients with an established diagnosis but without risk factors and for individuals with a positive genotype without phenotypic traits after a case-by-case evaluation. The same guidelines state that, in the presence of a mild systolic dysfunction (ejection fraction between 45 and 54%) a stress echo during physical exertion should be performed, and if systolic function increases by less than 15%, a pathological condition should be considered.

## 10. Is Hypertrabeculation a Cardiomyopathy?

According to the latest ESC guidelines, cardiomyopathy is defined as “a myocardial disorder in which the heart muscle is structurally and functionally abnormal, in the absence of coronary artery disease (CAD), hypertension, valvular disease, and congenital heart disease (CHD) sufficient to cause the observed myocardial abnormality” [1]. In HT, this definition is completely fulfilled if patients present with a typical complication like ventricular dysfunction, cardioembolism, or arrhythmias. Nevertheless, the same guidelines state that HT should be considered a phenotypic trait rather than a cardiomyopathy in the general sense. In our opinion, in the absence of cardiac or extracardiac conditions capable of inducing an increase in trabecular presence, the possibility of cardiomyopathy must be accounted for. However, our recommendation is to approach every patient with HT based on the presence of an effective clinical syndrome rather than solely a morphological pattern.

The main criticism towards HT is that it could be an expression of different underlying conditions, i.e., considering it a step during the progression of another disease. One major aspect in favour of this opinion is the absence of a clear genetic pattern, since most patients do not seem to carry a specific mutation. Another relevant aspect is that, compared to other conditions, the presence of HT is frequently unrelated to overt disease, but it could be a functional adaptation to an increase in left ventricular preload. To date, several aspects of this condition could be explained by considering HT as a phenotypic expression of other pathological phenomena, but many problems with this interpretation persist. The high incidence of adverse events such as ventricular arrhythmias and the relative difficulty in predicting such occurrences are still matters of debate, and further light must be shed on these phenomena. In particular, the classical risk factors for the development of ventricular arrhythmias do not seem to be as valid in this disease. For instance, despite being considered a major indication for an ICD implantation, in such patients the reduction in LVEF has not been shown to increase the risk of arrhythmic phenomena. Such data are also confirmed by studies like the Danish trial [50], displaying a low incidence of ventricular arrhythmias in patients affected by HF in non-ischaemic cardiomyopathies. This could be a reflection of the different phenotypes of such patients, with some profiles manifesting a particular tendency towards the development of major arrhythmias rather than a reduction in systolic performance. Cardioembolism is another crucial issue; it carries a high incidence and, most importantly, high morbidity. No other cardiac disease, apart from ventricular akinesia due to previous myocardial infarction, is capable of inducing endoventricular thrombosis, and to date, we are not capable of predicting such an event. All these peculiar aspects seem to be more relatable to a unique condition rather than a phenotype shared by many different diseases, but further studies are needed to assess this theory. We still do not know if HT could be identified as an independent condition, but it could represent an early marker of other cardiomyopathies that should prompt a close follow-up. The uncertainty gravitating around HT and the relative absence of a definite pathway for such patients should induce a deeper and more thorough clinical investigation to prevent possible fatal complications.

## 11. Gaps in Evidence

Despite a wide range of literature encompassing several features of this disease, our knowledge of this condition is still unclear. First of all, it is still not defined if HT represents a cardiomyopathy per se or a phenotypic expression of other diseases with a poor outcome. In addition, the diagnosis requires a multiparametric approach, which is still not widely shared in the cardiological community, and an imprecise diagnostic process could hamper our knowledge of this cardiomyopathy by providing heterogeneous and misleading data. Furthermore, despite an impressive incidence of ventricular arrhythmias, there are no tools able for predicting such occurrences in patients affected by ET. Another important aspect is the problem of anticoagulation; the process leading to ventricular thrombus formation in this population seems to be unrelated to what happens in patients affected by other cardiac diseases, and thus, the appropriate pharmacological regimen and whether it should be introduced even as a primary prevention tool are still not known. In addition, there is no specific guideline addressing the various complications in patients with HT cardiomyopathy. Thus, a tailored approach is required, and case-by-case analyses should be performed, taking into consideration the phenotypic expression of the disease.

## 12. Conclusions

Over the years, our knowledge of this disease has completely changed, and its very recognition is now challenged. A plethora of studies have been made in characterising its clinical features and pathogenesis, but considering the high incidence of phenotypes and the enormous variability of diagnostic criteria, the data are still conflicting. In this article, we tried to shed some light on this obscure and unknown disease while providing a useful tool for approaching patients with HT in everyday clinical practice.
